# Recursive Training Strategy for a Deep Learning Network for Segmentation of Pathology Nuclei With Incomplete Annotation

**DOI:** 10.1109/access.2022.3172958

**Published:** 2022-05-05

**Authors:** CHUAN ZHOU, HEANG-PING CHAN, LUBOMIR M. HADJIISKI, AAMER CHUGHTAI

**Affiliations:** Department of Radiology, University of Michigan, Ann Arbor, MI 48109, USA

**Keywords:** Deep learning, U-Net model, pathology images, image segmentation

## Abstract

This study developed a recursive training strategy to train a deep learning model for nuclei detection and segmentation using incomplete annotation. A dataset of 141 H&E stained breast cancer pathologic images with incomplete annotation was randomly split into training/validation set and test set of 89 and 52 images, respectively. The positive training samples were extracted at each annotated cell and augmented with affine translation. The negative training samples were selected from the non-cellular regions free of nuclei using a histogram-based semi-automatic method. A U-Net model was initially trained by minimizing a custom loss function. After the first stage of training, the trained U-Net model was applied to the images in the training set in an inference mode. The U-Net segmented objects with high quality were selected by a semi-automated method. Combining the newly selected high quality objects with the annotated nuclei and the previously generated negative samples, the U-Net model was retrained recursively until the stopping criteria were satisfied. For the 52 test images, the U-Net trained with and without using our recursive training method achieved a sensitivity of 90.3% and 85.3% for nuclei detection, respectively. For nuclei segmentation, the average Dice coefficient and average Jaccard index were 0.831±0.213 and 0.750±0.217, 0.780±0.270 and 0.697±0.264, for U-Net with and without recursive training, respectively. The improvement achieved by our proposed method was statistically significant (*P* < 0.05). In conclusion, our recursive training method effectively enlarged the set of annotated objects for training the deep learning model and further improved the detection and segmentation performance.

## I. INTRODUCTION

The success of deep learning (DL) models in automating a wide variety of tasks, e.g., image recognition, speech recognition, object detection and recognition, not only rely on the advancements in the model structures but also the development of learning algorithms and strategies [1]. There are strong correlations between the capability of effective training of the DL models, the number of model parameters, and the size of the training data [2], [3]. The DL models usually are configured with a large number of parameters to characterize complex patterns in real world data. To train these models, a large amount of data with clean annotations as reference standards are required [4]. However, the process of acquiring the annotations is long, laborious and costly. In medical field, it becomes even more difficult because it requires highly qualified professionals to provide the annotations. Automated image segmentation plays an essential role in developing computer-aided detection or diagnosis systems in medical imaging. For a task of identifying/detecting a specific object in the images, it is feasible to train a DL model with weak annotation, such as using a bounding box to enclose the object or a single point to mark the object location. However, in order to train a DL model to segment an object accurately from its background, it usually requires pixel-by-pixel outlining of a large number of objects as the training samples. When a large number of target objects need to be annotated for each image, it is difficult to make a full annotation even for a small number of cases [5]. Studies showed that, a typical whole slide digital pathology (WSDP) image with a size of 100,000 × 100,000 pixels may contain about 700,000 nuclei [6], [7]. It is nearly impossible for pathology professionals to manually outline the numerous nuclei in each image.

A substantial number of studies have developed image segmentation methods using various deep learning models in a wide range of applications [6], [8]-[16]. The availability of a large, well-annotated data set is often the limiting factor for the training and utilization of deep learning models for a given task. Transfer learning from natural images such as the big dataset of ImageNet has been widely adapted for medical image classification [2], [3], [6], [7]. The power of transfer learning, as well as data fusion, weight regulation and data augmentation, can be leveraged to fine-tune a pre-trained deep learning model with a relatively small data set, which have been shown to be useful to improve training effectiveness [10]. Despite how well those methods would perform, when transferring the pre-trained model (with natural images) to less correlated data (such as pathological images), transfer learning still needs extensive efforts in tuning network structures and optimizing the factors that may impact the model performance. In addition, the target objects in the training images are usually required to be fully annotated.

To our knowledge, the current publicly available data sets with nuclei fully annotated [17] are Multi-Organ Nucleus Segmentation (MoNuSeg) challenge [18] and Triple Negative Breast Cancer (TNBC) dataset [19]. The MoNuSeg dataset contained 44 sub-images with a size of 1000 × 1000 pixels cropped from hematoxylin and eosin (H&E) stained WSDP images at 40× magnification. The TNBC dataset included 50 sub-region images (512 × 512 pixels) cropped from 11 patients. The largest available dataset to-date was from a study that applied a resolution adaptive deep hierarchical (RADHicaL) scheme to nuclei segmentation [11]. The dataset contained 141 H&E images with a size of 2000 × 2000 pixels scanned at 40× magnification from 137 patients. However, the nuclei in each image were not fully annotated across the entire dataset.

Few studies have tackled the problem of incomplete annotation in the training of DL models for image segmentation. In particular, learning with incomplete annotations in WSDP images for cell segmentation has not been adequately explored [17]. Because it is difficult to create fully annotated WSDP images for a large number of cases, making use of available incompletely annotated datasets, such as RADHicaL, can be a useful alternative if an effective learning method can be developed. In this study, we proposed a recursive learning strategy to train deep learning models using incomplete annotations. Our primary focus was to develop a practical method that can facilitate the use of available incomplete annotation data to improve the training of deep learning models [20]. We used U-Net for the task of nuclei segmentation in pathologic images as an example. To evaluate the effectiveness of our proposed method, we compared the trained U-Net with and without recursive training method using an independent test set held out from the available data set. We also compared our trained model with the state-of-art methods reported in the literature using a public test dataset. The major contribution of this work is that our recursive approach provides a relatively simple and practical framework to tackle the problem of incomplete annotation. This framework does not depend on the specific network structure used and thus has the potential to be utilized for training other networks when fully annotated data are not available.

## II. MATERIALS AND METHODS

### A. DATA SET

**FIGURE 1.**
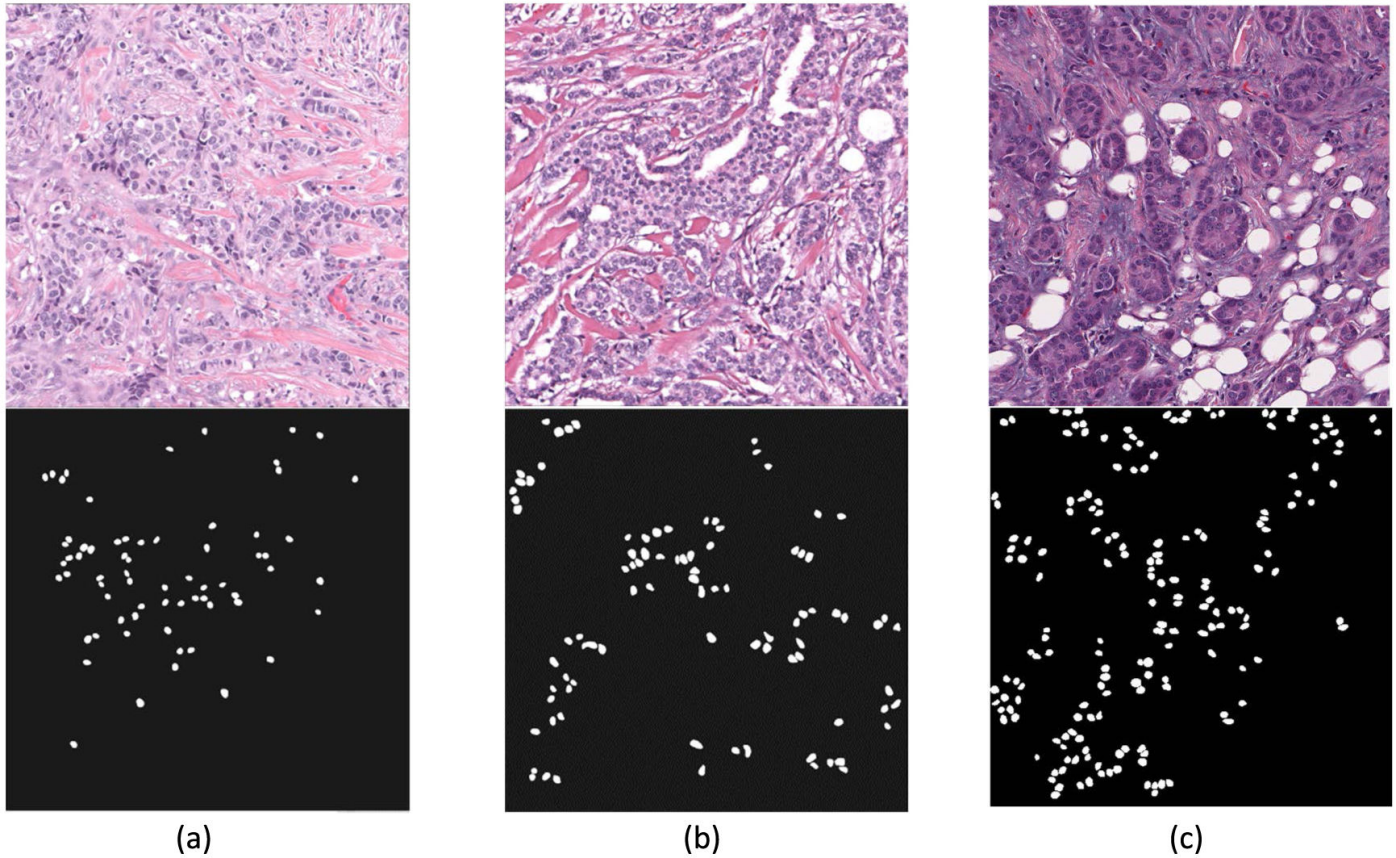
Examples of three 2000 × 2000-pixel H&E stained breast cancer (ER + BCa) histology images (upper row) with incomplete annotations (lower row) in the training set, containing 67, 89 and 173 annotated nuclei in column (a), (b) and (c), respectively.

A publicly available dataset (RADHicaL) was used in this study. The dataset contained H&E stained estrogen receptor positive (ER+) breast cancer (ER+ BCa) histology images [11], [21]. The WSDP images from 137 patients were scanned at 40x magnification, and 141 images with a size of 2000 × 2000 pixels were cropped from the WSDP images. A total of 12,726 nuclei were manually outlined in the 141 images and provided with the image set as reference standard. As manual annotation of individual nuclei on the histology images is laborious and time consuming, none of the images came with a complete annotation for all nuclei. Figure 1 shows examples of three 2000 × 2000-pixel image with incomplete annotation. In our study, we randomly split the 141 images on a per-patient basis into a training/validation set of 89 images from 85 patients and a test set of 52 images from 52 patients. A total of 8167 and 4559 nuclei were annotated in the 89 training and 52 test images with an average of 91.8 (range: 28 - 421, median: 66) and 87.7 (range: 44 - 342; median: 67) annotated nuclei per image, respectively.

### B. RECURSIVE DEEP LEARNING (R-DL) MODEL FOR NUCLEI DETECTION AND SEGMENTATION

**FIGURE 2.**
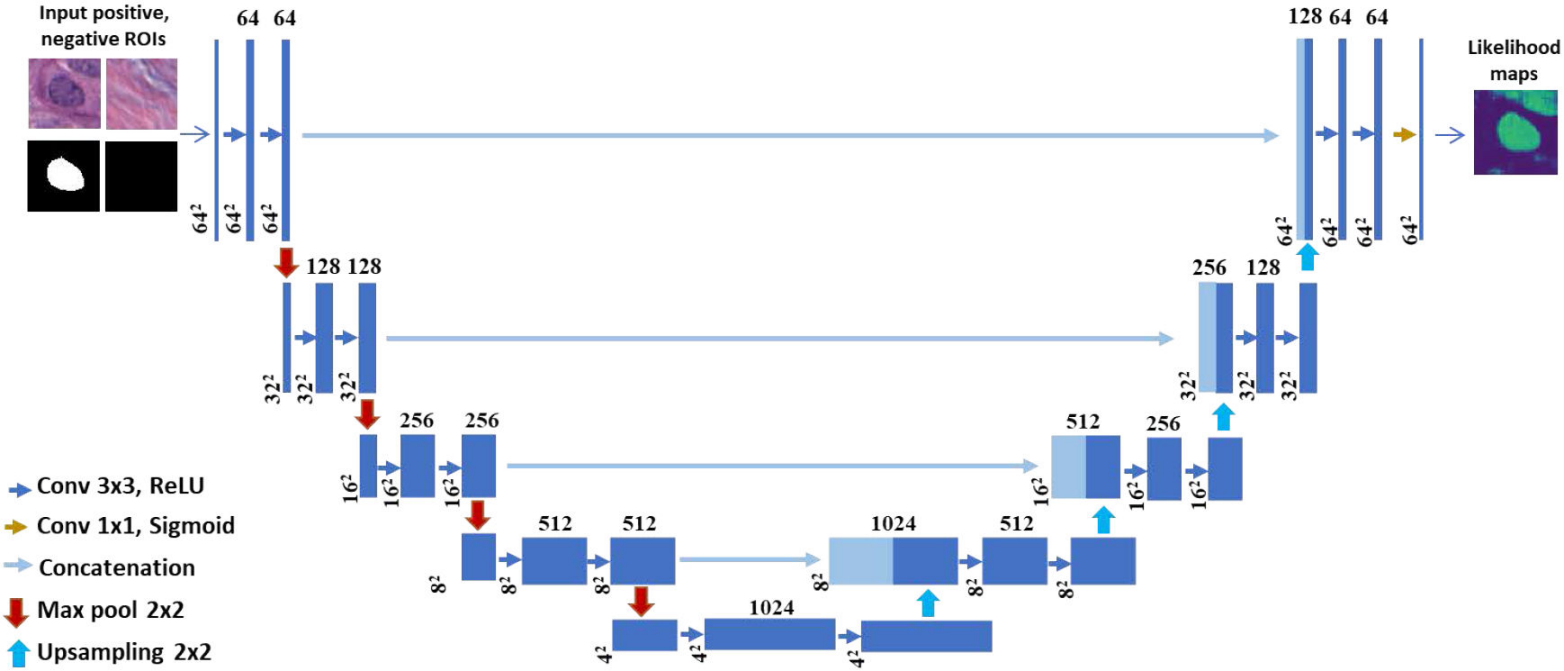
U-Net architecture as the backbone in recursive deep learning (R-DL) model for detection and segmentation of nuclei.

**FIGURE 3.**
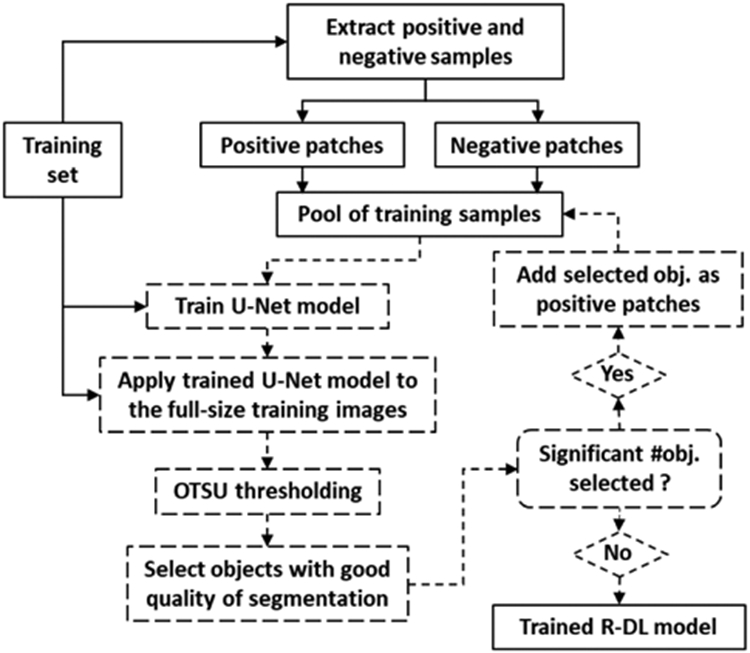
Schematic diagram of our method for training and test of a U-Net based recursive deep learning (R-DL) model for nuclei detection and segmentation. The recursive process was shown in the dash flowcharts.

We used the U-Net architecture (Figure 2) as backbone in our recursive deep learning (R-DL) model for detection and segmentation of nuclei in H&E stained digital histology images. The Pytorch python software package (version 1.2) with cuda toolkit 9.0 was used to implement the neural network. Figure 3 shows the schematic diagram of our R-DL method for training of a U-Net for nuclei detection and segmentation. To focus on studying the effectiveness of the recursive learning strategy rather than the design of the architecture of the DL model, without loss of generality, we retained the architecture and parameters of the conventional U-Net as much as possible. The U-Net consisted of three paths: the contracting, the bottleneck, and the expanding path. The contracting path was composed of 4 blocks to capture the context with multiresolution features. Each block consisted of two convolution layers (kernel size of 3 × 3) with stride 1 for extracting fine-grained image features, and a 2 × 2 max pooling operation with stride 2 for down-sampling. Each convolution layer was followed by a rectified linear unit (ReLU) activation function. The U-Net had 3 channels with the RGB image format as input. The 64 × 64-pixel image patch was input to the first block and started with 64 feature maps, the image was down-sampled to half size while the number of feature maps doubled after each block. The bottleneck of the network was built from two 3 × 3 convolution layers with dropout followed by a 2 × 2 up convolution layer to connect the contracting and expanding paths. Similar to the contracting path, the expanding path was also composed of 4 blocks. Each of the blocks consisted of a 1 × 1 upsampling layer with bilinear interpolation, a concatenation with the corresponding feature map from the contracting path followed by two 3 x 3 convolution layers (stride 1) with a ReLU activation function. At the end of the 4^th^ block in the expanding path, a 1 × 1 convolution layer with the sigmoid function was used to map the feature vector to the probability of being in the nucleus region for each pixel. Different from the contracting path, the image size was doubled by upsampling while the number of feature maps was reduced to half after each block.

### C. PREPARING THE TRAINING DATA

Because the true nuclei without annotations would be mistreated as the non-nuclei (mislabeled samples) background class if the incomplete annotation was used as reference to train a deep learning model, the missing annotations would likely cause erroneous predictions for both the true nuclei and non-nuclei classes. To limit the error of mislabeled samples propagating to the prediction, instead of using the entire images as training samples that contained both annotated and unlabeled nuclei, regions of interest (ROI) containing only annotated nuclei were extracted as positive training patches. The inclusion of only the labeled nuclei in the ROIs allowed the network to learn effectively from the positive patches for segmentation of nuclei. In a 2000 × 2000-pixel pathology image, 64 × 64-pixel ROIs were extracted, each was centered at an annotated nucleus to enclose the nucleus. Each ROI was augmented with affine translations by moving the ROI box with 16 pixels (1/4 of the ROI side-length) away from its center in the directions of up, down, left and right, resulting a total of 5 patches generated as positive training samples for each annotated nucleus. The 64 × 64 pixel patch size and the limited translation of 16 pixels allowed all patches to include information of the nucleus and some surrounding background for the network to learn image patterns. A total of 40,835 positive training/validation patches were generated from the 89 training/validation images.

**FIGURE 4.**
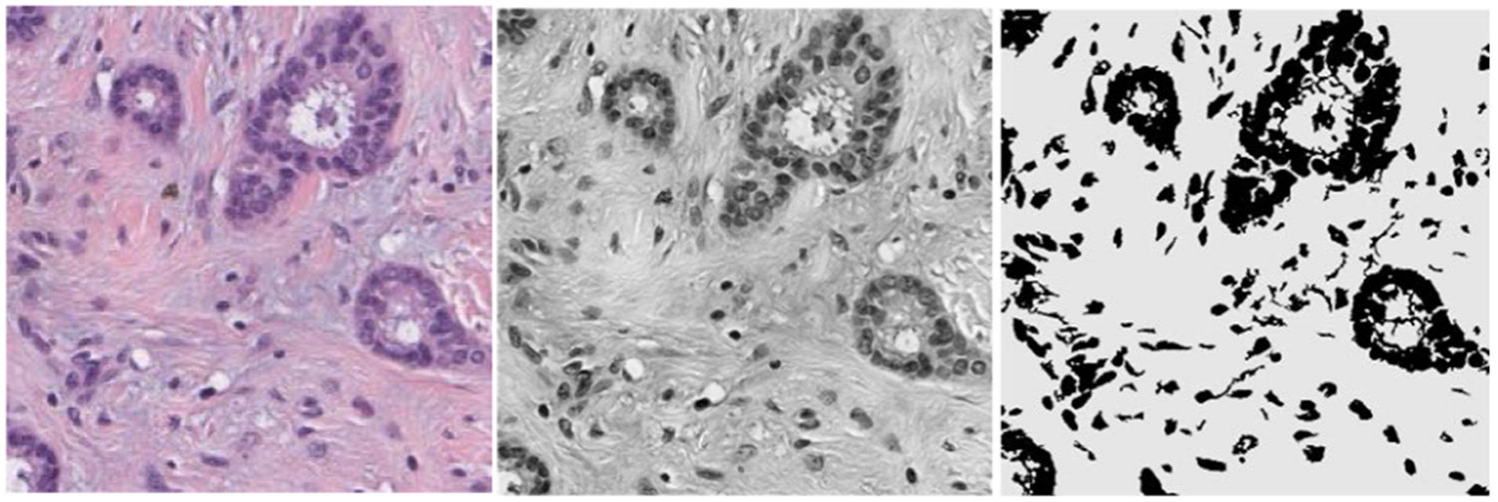
An example of negative regions (gray, right) estimated from thresholding the red channel (middle) of a 1000 × 1000-pixel sub-region of H&E image (left). Non-overlapping 64 × 64-pixel patches were randomly cropped from the non-nucleus regions as negative training samples.

As one pathologic image contains multiple nuclei, the detection of the nuclei locations will facilitate the segmentation task to delineate an accurate boundary or the region of each nucleus. To accomplish the task of simultaneous detection and segmentation in one model, we generated negative training patches from the training set images to characterize a wide variety of non-cell regions using a histogram-based semi-automatic method. The H&E staining produces colors for different tissue structures; generally the cell nuclei are stained in dark blue or purple and the extracellular materials are stained in red or pink. Based on the prior knowledge that the extracellular material depicts higher intensity than that of the nucleus regions in the red channel of H&E stained image (an example was shown in Fig.4), we developed a histogram-based thresholding method to extract the regions of extracellular material from a pathologic image. The red channel image was first read from the image file in RGB image format. Then an adaptive threshold *t* was calculated from the red channel image to segment the regions of extracellular material:

(1) t=a(μ∕σ)

where *μ* and σ was the mean and standard deviation of red channel image, and *a* was experimentally selected to be 0.5 to exclude lightly stained nuclei from regions with dark red. Finally, a morphologic dilation was performed to fill in the holes followed by a morphologic opening to remove small objects using a 20-pixel-radius disk-shaped structuring element. Figure 4 shows an example of segmented negative regions. We visually examined each segmented red channel images in the training set to crop and exclude the regions that contained nuclei. Non-overlapped 64 × 64-pixel patches were then randomly cropped from the examined regions. A total of 400,113 negative patches were generated. The negative patches combined with 40,835 positive patches formed a total of 440,948 training patches with approximately a 10:1 ratio of negative to positive patches.

### D. TRAINING THE R-DL MODEL

Recursive training of the R-DL model included three steps (Fig.3). In the initial step for configuration of training process, we randomly selected 10 validation cases from the training set, and the remaining 79 images from 75 cases with 36,239 positive and 355,102 negative patches were used as training samples. The U-Net was deployed on the 10 validation cases after each epoch. We monitored the learning by plotting the training loss and validation loss. Rather than optimizing the validation performance through fine-tuning the model parameters, we simply selected the training parameters such that the training process can perform from end-to-end with a decreasing trend for both the training and validation losses. The selected parameters included the learning rate, the weights of loss function, and batch size as well as the stopping criteria of the recursive process. A fixed number of 100 epochs was found to be sufficient in this initial training/validation step. Once the parameters were selected, at the second step the U-Net model was retrained using all patches from the 89 training images with 400 epochs. At the third step, we utilized our recursive training strategy to train the R-DL model using the whole set of patches from the 89 training images. After training was completed, the trained U-Net was deployed to the 52 independent test images for performance evaluation.

The U-Net backbone model was trained with a mini-batch stochastic optimization and the Adam algorithm (an extension of classical stochastic gradient decent algorithm) to improve the efficiency when about 400,000 patches were used for training samples. A mini-batch size of 4 patches was used and the mean gradient of the mini-batch was calculated to update the weights of the network in each epoch. We customized the loss function by combining a weighted Dice coefficient with binary cross-entropy (BCE):

(2) $$L=w_{1}L_{Dice}+w_{2}L_{BCE}$$

where *L* was the loss function, *w*_1_ and *w*_2_ were the weights, *L_Dice_* was the Dice loss derived from the Dice coefficient (also called *F*_1_ score) [22], *L_BCE_* was the BCE loss,

(3) $$L_{Dice}=1-\frac{2\sum_{i=1}^{N}p_{i}g_{i}+\varepsilon }{\sum_{i=1}^{N}p_{i}+\sum_{i=1}^{N}g_{i}+\varepsilon }$$

(4) $$L_{BCE}=-\frac{1}{N}\left(\sum_{i=1}^{N}(g_{i}\;\log\;(p_{i})+(1-g_{i})\;\log(1-p_{i})\right)$$

where *N* was the number of pixels in the image, *g_i_* ϵ {0, 1} was the annotation of the *i_th_* pixel, where 0 and 1 represented the pixel in the background and nucleus regions, respectively. 0 ≤ *p_i_* ≤ 1 was the probability of the *i_th_* pixel predicted being in the nucleus regions. The *L_Dice_* was also known as the soft Dice loss as it used the predicted probabilities instead of thresholded binary values. Small nuclei (such as the normal cells) may occupy fewer pixels and cause numerical instability in the *L_Dice_*. With the use of *L_Dice_* as the major component in the loss function to assess the segmentation performance and work with the imbalanced data between segmentation classes, we combined the *L_BCE_* with *L_Dice_* to reduce the numerical instability that might happen in *L*_Dice_. Experimentally, by using the training and validation sets, we selected the parameters, *ε* = 1, *w*_1_ = 1, and *w*_2_ = 0.1 to control the numerical stability and the balance between the two classes.

After the U-Net model was retrained in the second step using the 89 training images with 400 epochs, which was referred to as the training of the initial deep learning (I-DL) model, the model structure, weights and all other parameters of the trained I-DL were saved to a Python pickle utility file. The I-DL model was then set to the evaluation mode and loaded with the saved weights, and applied to each image in the training set in the inference process. The full resolution pathologic images with 2000 × 2000 pixels in RGB channels were input to the I-DL model. For each input pathologic image, the I-DL output a nuclei likelihood map in which each pixel indicated the likelihood of the pixel being in the nucleus regions. An Otsu automatic thresholding method [23] was used to segment the likelihood map into a binary image with nuclei and background regions. A morphological opening operation with a disk-shaped structuring element 5 pixels in radius was then used to remove the small objects from the segmented binary image. For each object segmented from the likelihood map images, we designed the following features as the quality indicators to extract the objects with high quality of segmentation:

(*f_1_*) Roundness (*Rdn*)

(5) $$Rdn=4\pi A∕P^{2}$$

where *A* and *P* are the area and perimeter of the segmented object.

(*f_2_*) Solidity (*S*)

(6) S=A∕CA

where the *CA* is the area of the convex hull [24] of the segmented object.

The segmented object with *f*_1_ > 0.7 and *f*_2_ > 0.9 was determined to be a high quality nucleus. With f_1_ and f_2_ as the quality indicators, a set of segmented nuclei was selected. The same procedure of augmentation was applied to each newly selected nucleus to generate five 64 × 64 patches by cropping ROIs at and around the center of each nucleus. The augmented patches were then added to the pool of previously generated positive training samples, forming a new set of positive training samples. As the negative training samples had been visually examined to exclude any nuclei, there would be no change in the set of the negative training samples in the recursive training. Using the training samples that combined the newly generated positive samples with the negative samples, the U-Net model was retrained with 400 epochs. The above process was recursively repeated until no significant number of high quality nuclei could be selected. As the recursive training progressed, the number of newly generated positive samples would decrease and the impact on the model accuracy also diminished. Thus it is useful to set an early termination criterion to control the balance between the computational cost and the improvement of model accuracy during the recursive process. By using the training set and validation set, we experimentally selected a threshold of 20% such that if the additional number of high quality nuclei was less than 20% of the current number of positive samples, then the recursive process would be terminated.

### E. PERFORMANCE EVALUATION

The DL performance in simultaneous nuclei detection and segmentation was evaluated by comparing the segmentation results to the manually outlined nuclei as the reference standard. The performances of the R-DL and I-DL were compared on the independent test set of 52 images. The sensitivity of nuclei detection and the Dice coefficient and Jaccard index for nuclei segmentation were used as quantitative performance measures. We used an overlap criterion to determine whether a reference standard nucleus was detected by the DL model. A reference standard nucleus was marked as detected when the overlap ratio between a DL segmented object and the reference standard nucleus was greater than a threshold *T*. The overlap ratio *R_JI_* was defined as the Jaccard index of similarity measure (also called intersection over union (IoU)) [25]:

(7) $$R_{JI}=\frac{Obj\cap Ref}{Obj\cup Ref}$$

where *Obj* was the segmented object, *Ref* was the reference standard nucleus. When a reference standard nucleus overlapped with more than one object, the object with the largest overlap ratio among the multiple overlapping objects was counted. The threshold *T* was chosen to be 50% in this study, which also indicated that the segmented object had 50% similarity with the reference standard nucleus. The sensitivity of nuclei detection was thus defined as the number of detected reference standard nuclei versus the total number of reference standard nuclei.

For a detected object that had an overlap ratio *R_JI_* >= 0.5 with a reference standard nucleus, the Dice coefficient was used to assess the segmentation accuracy for that object:

(8) $$Dice=\frac{2(Obj\cap Ref)}{Obj+Ref}$$

The average Dice coefficient and the average Jaccard index were calculated as quantitative measures to assess the segmentation accuracy for all segmented objects that had overlap ratio *R_JI_* >= 0.5 with reference standard nuclei in the 52 test images.

## III. RESULTS

**FIGURE 5.**
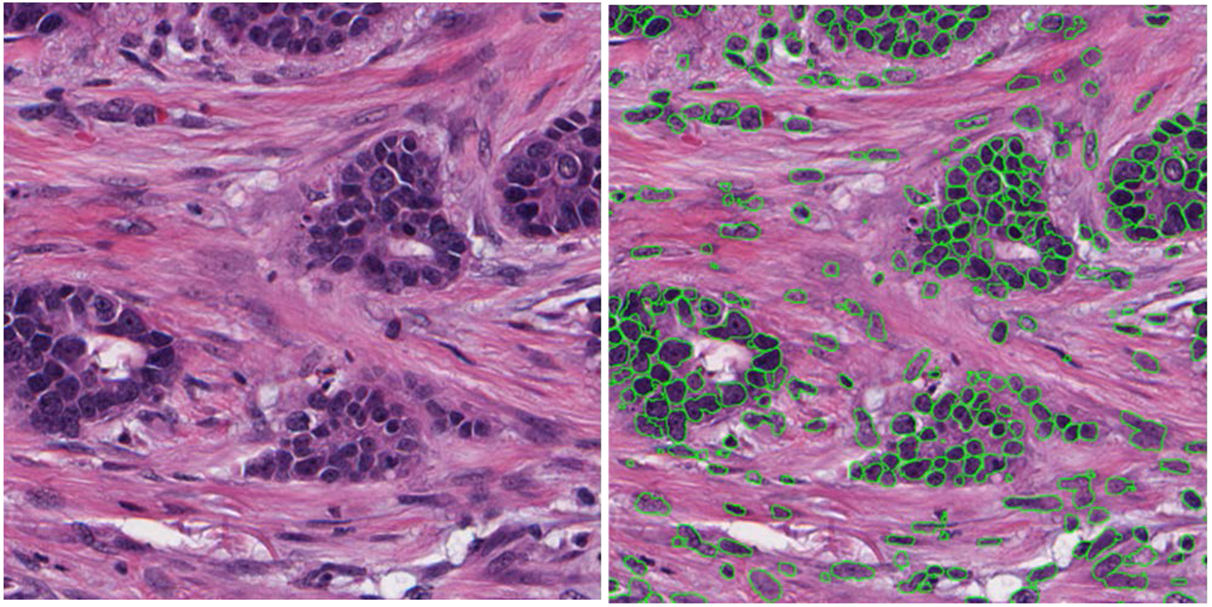
An example of segmented nuclei by the R-DL model trained with our recursive training method. Left: an enlarged 1000 × 1000-pixel sub-region from the original 2000 × 2000-pixel H&E image in the test set. Right: The segmented nuclei enclosed with green contours.

For the 52 test images with a total of 4559 annotated nuclei, our R-DL model achieved a sensitivity of 90.3% (4117/4559) for nuclei detection. The average Dice coefficient and average Jaccard index were 0.831±0.213 and 0.750±0.217, respectively. Figure 5 shows an example of segmentation result by the R-DL model.

Compared to the R-DL model with recursive training, the I-DL model without recursive training achieved a sensitivity of 85.3% (3891/4559) for nuclei detection. The average Dice coefficient and average Jaccard index for nuclei segmentation by the I-DL model were 0.780±0.270 and 0.697±0.264, respectively. The R-DL model with the proposed recursive training strategy achieved significant improvement compared with the I-DL model (*P* < 0.05 by two-tailed paired *t*-test).

**FIGURE 6.**
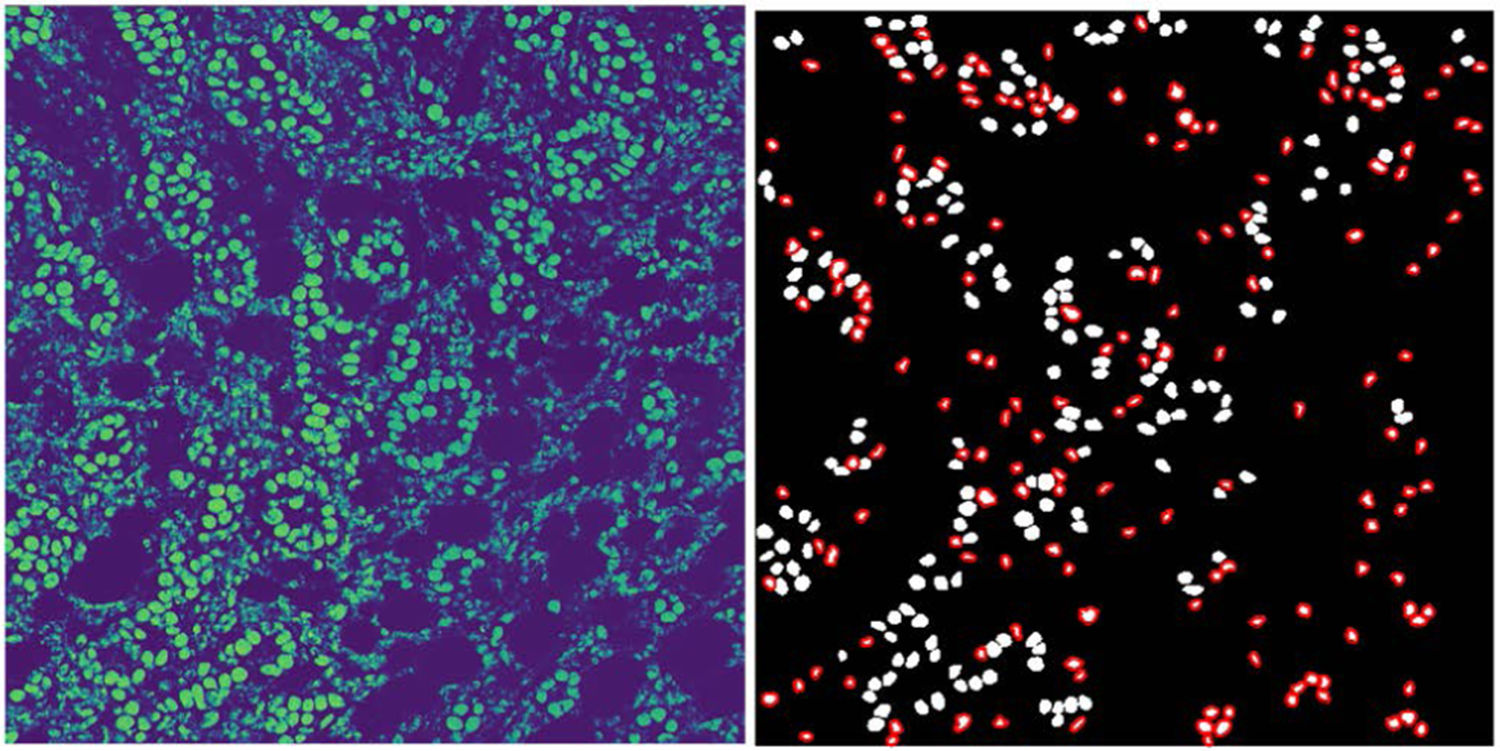
Results of the I-DL model segmentation when the I-DL model trained using manually outlined nuclei was applied to the training image shown in Fig.1(c). Left: likelihood map output from the model. Right: new set of positive nuclei formed by combining a set of selected high quality segmentation of objects (red enclosed) with the manually outlined nuclei (white).

**FIGURE 7.**
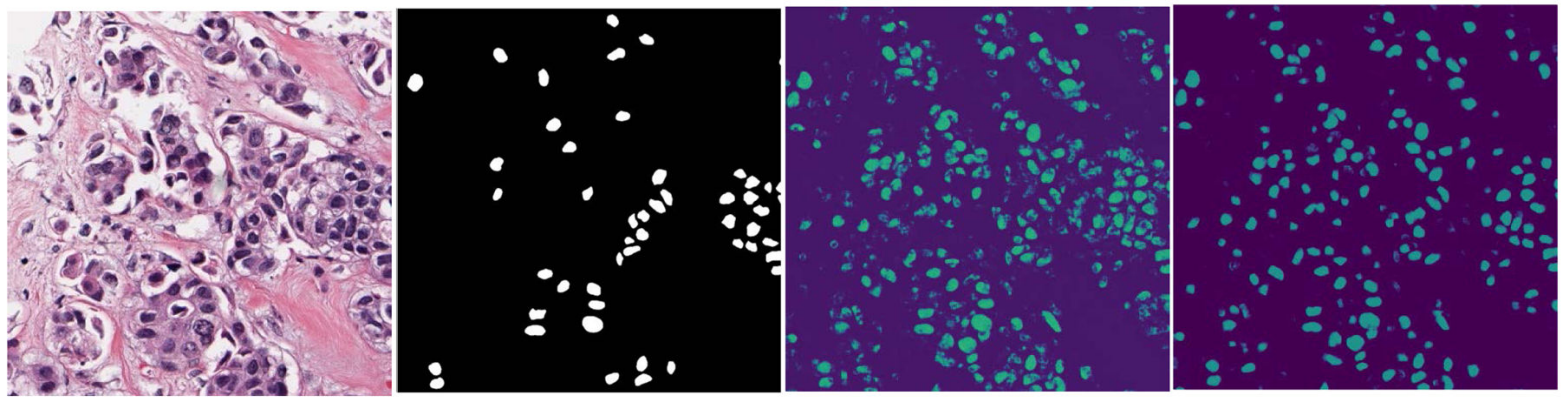
Examples of the likelihood maps output from the U-Net model with and without using our recursive training method. 1^st^ column: an enlarged 1000 × 1000-pixel sub-region from the original 2000 x 2000-pixel H&E stained image in the test set; 2^nd^ column: manually outlined incomplete annotation of nuclei; 3^rd^ columns: likelihood map output from the I-DL model without recursive training; 4^th^ column: likelihood map output from the R-DL model with recursive training.

Figure 6 shows an example of the likelihood map output from the I-DL model without recursive training when the trained I-DL model was applied to a training image, and a set of high quality objects (enclosed in red contours) selected from the thresholded likelihood map as positive training nuclei which was combined with the manually outlined nuclei (white) to be used for the next round of recursive training. An example of the improved performance of the U-Net obtained with our recursive training method is shown in Fig. 7. It clearly shows that the likelihood map output from the I-DL model contained a lot of noise while the nucleus regions in the likelihood map output from the R-DL model become more solid and noise in the background was largely reduced.

Table 1 shows the performance comparisons between our R-DL model and five different methods reported in the literature [17]. This published study [17] reviewed and compared various methods for segmentation of breast tumor nuclei, including DL networks of U-Net like models (i.e. VGG, ResNet and DenseNet)[26], Ensemble U-Net [17], Mask R-CNN [27], GB U-Net [17] and a classical method of Fiji [28](an open-source software package features a built-in nuclei segmentation pipeline). With the MoNuSeg dataset, the reported DL networks were trained and tested with 36 and 8 H&E images (1000 × 1000 pixels), respectively, in which the nuclei were fully annotated. Without any retraining and modification, we deployed our trained R-DL model directly to the MoNoSeg test set containing 8 breast tissue images (Table 1). The test results showed that, the average Aggregated Jaccard Index (AJI) [29] score of 0.475 achieved by our R-DL model was significantly higher than that of 0.340 achieved by the Fiji method (P<0.05), and was not significantly different from those of the U-Net based DL models (P>0.05), such as DenseNet-201 U-Net, Ensemble U-Net and GB U-Net. The AJI by our R-DL was significantly (P<0.05) lower only compared to that of the Mask R-CNN that used pre-trained ResNet-101 as the backbone. In addition to the AJI metric and the models showed in Table 1, Table 2 shows the results of our DL models with and without recursive training, and other U-Net like models reviewed in the literature [17], with respect to different evaluation metrics including mean average precision (mAP) over ten IoU thresholds increasing linearly from 0.5 to 0.95 by 0.05, and the metrics of F1, recall and precision with IoU thresholds of 0.5 and 0.7, respectively. The results showed that, our R-DL model achieved a higher mAP of 0.3538 than all reviewed models except Mask R-CNN and GB U-Net. Furthermore, our R-DL scored the highest at an IoU threshold of 0.7 in F1, recall and precision (except Mask R-CNN and GB U-Net), but scored the lowest (except Fiji) at an IoU threshold of 0.5 in F1, recall and precision, indicating that the performance of the proposed R-DL was comparable to methods reported in the literature.

## IV. DISCUSSION

With the increased access to data (“big data”), open-sourced software frameworks, and accelerating computation with powerful hardware, deep neural networks are now the state-of-the-art machine learning models with a wide variety of applications from image analysis to natural language processing. However, annotated data are required for efficient supervised learning but data with good annotation are limited. In particular, the training of deep learning neural networks for image segmentation remains challenging not only because of the very high dimensionality of their parameter space but also the requirement of a large amount of training samples with clean annotation in which the object boundaries need to be outlined. In this study, we proposed a new recursive training strategy to train a deep learning model with incomplete annotation and demonstrated the effectiveness of our proposed method by training a U-Net model to detect and segment nuclei in H&E stained pathologic images. Among the wide variety of deep learning neural network models available for image segmentation, e.g., convolutional neural networks (CNNs), fully connected networks (FCNs), encoder-decoder model, and recurrent neural networks (RNNs), we chose U-Net as a representative model because it is a popular deep learning model that was initially developed for biomedical image segmentation inspired by CNNs, FCNs and encoder-decoder models, and has been widely used in the field of medical imaging. The primary focus of our study is on how to effectively train a deep learning model using available incomplete annotation. Without loss of generality, we kept the conventional U-Net architecture basically unchanged so that the readers will not be distracted by the design of the CNN architecture and focus on the merit of our proposed recursive training method. This not only facilitates comparisons of our method with the conventional U-Net and other methods, but also provides flexibility that other network architectures including pre-trained networks (with transfer learning) can be used as backbones for nuclei or other image segmentation tasks because the U-Net is well-understood and the replacement or comparison of U-Net with many other CNN architectures has been extensively studied in the literature. As can be seen from the comparisons in Table 1 and Table 2, even with a commonly used U-Net, the recursive training could bring its performance to be comparable to those of the more complicated networks trained with fully annotated samples. Since the recursive training framework is not specific to the network architecture, it can be expected that any other networks can be used in place of the U-Net and gain similar advantage of increased training sample size by our proposed approach.

There are three key issues involved in the training of our R-DL model: the training sample presentation, the initial training of the backbone DL model, and the selection of the nuclei candidates as positive training samples based on their segmentation quality in the recursive loops. As the annotation of nuclei is a time consuming task, only a small portion of nuclei could be manually outlined in an image. If the entire image that contains true nuclei both with and without outline is used to train the network, those numerous unlabeled true nuclei will be treated as negatives thus providing contradictory information with those labeled positives during network learning. To reduce the data bias, we extracted the ROI centered at each outlined nucleus as a training sample patch. In general, the cancer cell nuclei differ from normal nuclei in their enlarged sizes and deformed spheroid shapes due to their invasion into nearby tissues. With prior knowledge of the size distribution of the outlined cancer and normal nuclei in 40x magnified H&E ER + BCa pathologic images, the average long-axis diameter of cancer nucleus is about 32 pixels. We used a fixed 64 × 64-pixel ROI as positive training samples that enclosed each outlined nucleus and its surrounding background. As the edges and topologic information of nuclei may play important roles in separating connected cells, in addition to use a relatively large ROI to include the background surrounding the nucleus and other nearby nuclei, we also included augmented ROIs by affine translation. To facilitate nuclei detection simultaneously with nuclei segmentation, we used a semi-automatic method to extract a set of negative patches from the red channel of H&E stained images based on the biologic characterization that the H&E stains the majority of the cell nuclei and extracellular materials in different colors. Due to inconsistencies in laboratory protocol of H&E staining that may cause color variations, we visually excluded the regions that contain nuclei after the initial automatic segmentation. Compared with outlining nuclei contours, the visual examination was a relatively easy task which was done by using an interactive graphical user interface.

In the recursive loops of R-DL training, the selection of nuclei with high segmentation quality was a critical step. We designed morphologic metrics to characterize the high quality of segmented nuclei as relatively round and dense objects in the segmented images. With the training/validation set, we designed a rule-based method by adjusting the thresholds and visually examining the selection results. We found that the high quality segmented nuclei could be selected efficiently using our rule-based method and were useful for improving network training when they were fed back to the R-DL model during recursive training. In our experiments, the average training time of 100 epochs for this application was 5.1 hours on an NVIDIA RTX 2080 Ti GPU with cuDNN v3 acceleration. We used an early termination criterion as the number of high quality nuclei to be gained diminished as the recursive iteration progressed, and was able to limit the recursive training to less than or equal to 3 iterations without significant loss of model accuracy. Further study is warranted to investigate the impact of selected nuclei with a range of quality as well as optimization and automation of the selection method within the recursive training loop. That the recursive training is not fully automated is one of limitation of our current study. Another limitation of our study is that we evaluated the detection and segmentation performance using only the annotated nuclei as reference standard. As shown in the examples in Figures 6 and 7, a large number of true nuclei were detected and segmented beyond the annotated nuclei. Due to the fact that the reference standard came as a part of the dataset, we could only include the labeled nuclei in the calculation of sensitivity of nuclei detection, and not able to credit those detected unlabeled nuclei. We were also not able to evaluate the segmentation accuracy of those unlabeled nuclei and the true and false positive fractions in nuclei detection. Qualitatively, it can be seen in the example in Fig. 7 that the R-DL model substantially enhanced the true nuclei regions compared to the I-DL model.

Comparing with the state-of-art methods for nuclei segmentation that were reviewed and evaluated by literature [17], although our R-DL model was trained with the incomplete nuclei annotation, the performance achieved by our R-DL model was competitive to those DL models that were trained with fully annotated nuclei images. Additionally, those DL models either utilized more advanced network structures, such as DenseNet or ResNet, as the backbone, or combined different U-Net like networks in DL models, such as Ensemble U-Net and GB U-Net, to improve the accuracy of nuclei segmentation. The limitation of both our and the study in the literature [17] was using the relatively small dataset for training and testing. We will further evaluate the detection and segmentation performances in future studies if large data sets with complete annotation become available.

## V. CONCLUSION

We have developed a recursive training method to train a U-Net deep learning model for simultaneous detection and segmentation of nuclei in H&E stained ER+ BCa histology images with incomplete annotation. Our methods that selectively chose positive and negative patches from incompletely annotated pathologic data can be used to train the U-Net model to detect and segment nuclei with high accuracy. The recursive training method effectively increased the training samples to train the DL model and significantly improved the detection and segmentation performances.

## Figures and Tables

**TABLE 1.** Performance comparison between our R-DL method and literature evaluated methods. Individual Aggregated Jaccard Index (AJI) scores were obtained from eight breast tissue images from MoNoSeg test dataset [17].

Image ID	Fiji	U-Net (DenseNet-201)	Ensemble U-Net	Mask R- CNN	GB U-Net	R-DL (proposed)
TCGA-A7-A13E-01Z-00-DX1	0.350	0.515	0.579	0.517	0.628	**0.465**
TCGA-A7-A13F-01Z-00-DX1	0.328	0.434	0.491	0.554	0.518	**0.503**
TCGA-AC-A2FO-01A-01-TS1	0.252	0.493	0.478	0.543	0.511	**0.448**
TCGA-AO-A0J2-01A-01-BSA	0.323	0.561	0.486	0.56	0.535	**0.519**
TCGA-AR-A1AK-01Z-00-DX1	0.31	0.526	0.458	0.511	0.5	**0.395**
TCGA-AR-A1AS-01Z-00-DX1	0.634	0.623	0.632	0.611	0.676	**0.528**
TCGA-E2-A14V-01Z-00-DX1	0.272	0.482	0.436	0.5	0.464	**0.487**
TCGA-E2-A1B5-01Z-00-DX1	0.249	0.433	0.379	0.43	0.432	**0.452**
TCGA-A7-A13E-01Z-00-DX1	0.35	0.515	0.579	0.517	0.628	**0.465**
Average AJI	0.340	0.508	0.493	0.528	0.533	**0.475**
P-value *	0.0093	0.1764	0.5003	0.0113	0.0585	

* P-value (by paired t-test) of the differences between our R-DL model and other five methods.

**TABLE 2.** Comparison between our R-DL and I-DL methods with and without recursive training and literature evaluated methods with respect to different evaluation metrics. The metrics were obtained from the MoNoSeg test set containing 8 breast tissue images [17]. The mean average precision was calculated with ten IoU thresholds from 0.5 to 0.95, while the metrics of F1, recall and precision were reported with IoU thresholds of 0.5 and 0.7, respectively.

Segmentation Methods	AJI	Mean Avg. Precision	F1 (0.7)	Recall (0.7)	Precision (0.7)	F1 (0.5)	Recall (0.5)	Precision (0.5)
Fiji	0.3396	0.2370	0.1828	0.1447	0.2481	0.4411	0.3493	0.5986
U-Net(VGG-16)	0.4925	0.2736	0.2886	0.2927	0.2845	0.6511	0.6604	0.642
U-Net(VGG-19)	0.4841	0.3007	0.3452	0.348	0.3426	0.6735	0.6788	0.6683
U-Net(ResNet-50)	0.4882	0.3163	0.3772	0.3884	0.3667	0.6967	0.7173	0.6772
U-Net(ResNet-101)	0.4687	0.3318	0.3242	0.2834	0.3788	0.6133	0.536	0.7166
U-Net(ResNet-152)	0.4396	0.3119	0.3368	0.3241	0.3506	0.6706	0.6452	0.698
U-Net(DenseNet-121)	0.4668	0.2988	0.3579	0.3738	0.3432	0.6796	0.7099	0.6517
U-Net(DenseNet-201)	0.5083	0.3185	0.376	0.3884	0.3645	0.698	0.7208	0.6765
U-Net(Inception-v3)	0.444	0.2879	0.3044	0.3005	0.3085	0.6422	0.6339	0.6507
Mask R-CNN	0.5282	0.3884	0.4028	0.3518	0.4773	0.6648	0.5813	0.7859
U-Net Ensemble	0.4926	0.3381	0.3791	0.3677	0.3913	0.6957	0.6746	0.718
GB U-Net	0.5331	0.3909	0.4007	0.3509	0.4669	0.6862	0.601	0.7997
**R-DL (proposed)**	**0.4746**	**0.3538**	**0.4211**	**0.3914**	**0.4659**	**0.5566**	**0.5169**	**0.6171**
**I-DL (w/o recursive)**	**0.4044**	**0.2710**	**0.2731**	**0.2386**	**0.3354**	**0.4291**	**0.3743**	**0.5293**

## References

[1] LeCun Y, Bengio Y, and Hinton GE, “Deep learning,” Nature, vol. 521, pp. 436–444, Dec. 2015. 26017442 10.1038/nature14539

[2] Goodfellow I, Bengio Y, and Courville A, Deep Learning. Cambridge, MA, USA: MIT Press, 2016.

[3] Samala RK, Heang-Ping C, Hadjiiski L, Helvie MA, Richter CD, and Cha KH, “Breast cancer diagnosis in digital breast tomosynthesis: Effects of training sample size on multi-stage transfer learning using deep neural nets,” IEEE Trans. Med. Imag, vol. 38, no. 3, pp. 686–696, Mar. 2019. 10.1109/TMI.2018.2870343 PMC6812655 31622238

[4] Esteva A, Kuprel B, Novoa RA, Ko J, Swetter SM, Blau HM, and Thrun S, “Dermatologist-level classification of skin cancer with deep neural networks,” Nature, vol. 542, no. 7639, pp. 115–118, 2017. 28117445 10.1038/nature21056 PMC8382232

[5] Armato SG, McNitt-Gray MF, Reeves AP, Meyer CR, McLennan G, Aberle DR, Kazerooni EA, MacMahon H, van Beek EJ, Yankelevitz D, and Hoffman EA, “The lung image database consortium (LIDC): An evaluation of radiologist variability in the identification of lung nodules on CT scans,” Academic Radiol., vol. 14, no. 11, pp. 1409–1421, Nov. 2007. 10.1016/j.acra.2007.07.008 PMC2290739 17964464

[6] Sadanandan SK, Ranefall P, Guyader SL, and Wählby C, “Automated training of deep convolutional neural networks for cell segmentation,” Sci. Rep, vol. 7, no. 1, p. 7860, Aug. 2017. 28798336 10.1038/s41598-017-07599-6 PMC5552800

[7] Wahab N, Khan A, and Lee YS, “Transfer learning based deep CNN for segmentation and detection of mitoses in breast cancer histopathological images,” Microscopy, vol. 68, no. 3, pp. 216–233, Jun. 2019. 30722018 10.1093/jmicro/dfz002

[8] Arbelle A and Raviv TR, “Microscopy cell segmentation via adversarial neural networks,” in Proc. IEEE 15th Int. Symp. Biomed. Imag. (ISBI), Apr. 2018, pp. 645–648.

[9] Araújo FHD, Silva RRV, Ushizima DM, Rezende MT, Carneiro CM, Bianchi AGC, and Medeiros FNS, “Deep learning for cell image segmentation and ranking,” Computerized Med. Imag. Graph, vol. 72, pp. 13–21, Mar. 2019. 10.1016/j.compmedimag.2019.01.003 30763802

[10] Tajbakhsh N, Jeyaseelan L, Li Q, Chiang JN, Wu Z, and Ding X, “Embracing imperfect datasets: A review of deep learning solutions for medical image segmentation,” Med. Image Anal, vol. 63, Jul. 2020, Art. no. 101693. 32289663 10.1016/j.media.2020.101693

[11] Janowczyk A, Doyle S, Gilmore H, and Madabhushi A, “A resolution adaptive deep hierarchical (RADHicaL) learning scheme applied to nuclear segmentation of digital pathology images,” Comput. Methods Biomech. Biomed. Eng., Imag. Vis, vol. 6, no. 3, pp. 270–276, 2018. 10.1080/21681163.2016.1141063 PMC5935259 29732269

[12] Yang L, Zhang Y, Chen J, Zhang S, and Chen D, “Suggestive annotation: A deep active learning framework for biomedical image segmentation,” in MICCAI, 2017, pp. 399–407.

[13] Kuo W, Häne C, Yuh E, Mukherjee P, and Malik J, “Cost-sensitive active learning for intracranial hemorrhage detection,” in Proc. MICCAI, 2018 pp. 715–723.

[14] Gal Y and Ghahramani Z, “Dropout as a Bayesian approximation: Representing model uncertainty in deep learning,” 2015, arXiv:1506.02142.

[15] Gorriz M, Carlier A, Faure E, and Giro-i-Nieto X, “Cost-effective active learning for melanoma segmentation,” 2017, arXiv:1711.09168.

[16] Zhang Y and Yang Q, “A survey on multi-task learning,” 2017, arXiv:1707.08114.

[17] Lagree A, Mohebpour M, Meti N, Saednia K, Lu F-I, Slodkowska E, Gandhi S, Rakovitch E, Shenfield A, Sadeghi-Naini A, and Tran WT, “A review and comparison of breast tumor cell nuclei segmentation performances using deep convolutional neural networks,” Sci. Rep, vol. 11, no. 1, p. 8025, Dec. 2021. 33850222 10.1038/s41598-021-87496-1 PMC8044238

[18] Kumar N, Verma R, Anand D, Zhou Y, Onder OF, Tsougenis E, Chen H, Heng PA, Li J, Hu Z, and Wang Y, “A multi-organ nucleus segmentation challenge,” IEEE Trans. Med. Imag, vol. 39, no. 5, pp. 1380–1391, May 2020. 10.1109/TMI.2019.2947628 PMC10439521 31647422

[19] Naylor P, Laé M, Reyal F, and Walter T, “Segmentation of nuclei in histopathology images by deep regression of the distance map,” IEEE Trans. Med. Imag, vol. 38, no. 2, pp. 448–459, Feb. 2019. 10.1109/TMI.2018.2865709 30716022

[20] Ronneberger O, Fischer P, and Brox T, “U-Net: Convolutional networks for biomedical image segmentation,” in Proc. Med. Image Comput. Comput.-Assist. Intervent (MICCAI), Cham, Switzerland, 2015, pp. 234–241.

[21] Janowczyk A and Madabhushi A, “Deep learning for digital pathology image analysis: A comprehensive tutorial with selected use cases,” J. Pathol. Inform, vol. 7, p. 29, Jul. 2016. 27563488 10.4103/2153-3539.186902 PMC4977982

[22] Sørensen TJ, A Method of Establishing Groups of Equal Amplitude in Plant Sociology Based on Similarity of Species Content and its Application to Analyses of the Vegetation on Danish Commons. København, Denmark: I kommission hos E. Munksgaard, 1948.

[23] Otsu N, “A threshold selection method from gray-level histograms,” IEEE Trans. Syst., Man, Cybern. Syst, vol. SMC-9, no. 1, pp. 62–66, Feb. 1979.

[24] Ye Q-Z, “A fast algorithm for convex hull extraction in 2D images,” Pattern Recognit. Lett, vol. 16, no. 5, pp. 531–537, May 1995.

[25] Jaccard P, “The distribution of the flora in the Alpine zone,” New Phytolog., vol. 11, pp. 37–50, Feb. 1912.

[26] Huang G, Liu Z, van der Maaten L, and Weinberger KQ, “Densely connected convolutional networks,” in Proc. IEEE Conf. Comput. Vis. Pattern Recognit, Jul. 2017, pp. 2261–2269.

[27] He K, Gkioxari G, Dollár P, and Girshick RB, “Mask R-CNN,” in IEEE Int. Conf. Comput. Vis. (ICCV), Oct. 2017, pp. 2980–2988.

[28] Schindelin J, “Fiji: An open-source platform for biological-image analysis,” Nature Methods, vol. 9, no. 7, pp. 676–682, Jul. 2012. 22743772 10.1038/nmeth.2019 PMC3855844

[29] Kumar N, Verma R, Sharma S, Bhargava S, Vahadane A, and Sethi A, “A dataset and a technique for generalized nuclear segmentation for computational pathology,” IEEE Trans. Med. Imag, vol. 36, no. 7, pp. 1550–1560, Jul. 2017. 10.1109/TMI.2017.2677499 28287963

